# 

**Published:** 1904-01-15

**Authors:** 


### ADDRESSES AND PAPERS.

Address of President, C. C. Noble, 487.
Address of President, J. P. Beauman, 352.
Adrenalin in Local Anesthesia, B. B. Atchison, 517.
Allays of Commerce and their Metal Formulae, N. K. Garhart, 217.
Aluminum, E. Bumgardner, 193.
Amalgam, H. P. Martin, 109.
Care of the Oral Cavity During Pregnancy, C.H.Oakman,163.
Comparison of Jenkins and High Fusing Bodies, J. Q. Byram, 271.
Compressed Air in Dentistry, Geo. Zederbaum, 541.
Dental Book-keeping, H. T.Smith, 385.
Doctor Analyzed, J. W. Clemmer, M. D., 393.
Enamel Walls and Margins, L. P. Bethel, 433.
Esthetic Dentistry Required, W. T. McLean, 347.
Ideal Dental Laboratory, C. H. Worboys, 595.
Illogical Terminology, E. T. Loeffler, 490.
Impressions for Orthodontia, B. Abell, 499.
Inlay Cavity Preparation, F. E. Roach, 217.
Inlay Matrix Management, L. E. Custer, 282.
Mat Gold and How to Use It, J. L. Young, 471.
Mid-winter National Meetings, N. S. Hoff, 1.
Models for Orthodontia, M. T. Watson, 496.
Modern Operative Equipment, E. A. Honey, 601.
Obtunding Dentine, F. C. Collins, 548.
Office Management, H. C. Raymond, 453.
Permanency of the Teeth, R. B. Howell, 338.
Practical Use of Nitrous Oxide, E. R. Jackson, 385.
Professional Standards, N. S. Hoff, 8.
Province of Porcelain in Dentistry, H. J. Goslee, 285.
Regulation of Fees, P. L. Hines, 462.
Seamless Crowns, M. L. Ward, 234.
Suppurative Peri-cementitis, A. B. Conklin, 166.
Syphilis of Dental Interest, D. M. Graham, 55..

### REPRINTS.

Address of President Charles Gordon, 509.
Adhesion of Vulcanite to Metals, Walter M. Bartlett, 613.
Adrenalin and Cocain, Fritz Hartwig, 175.
Cavity Preparation for Porcelain Fillings, W. T. Reeves, 555.
Correct Articulation in Prosthodontia, H. M. Kirk, 90.
Crowns, Improved Method of Making, Herbert Ziele, 95.
Cuspid Teeth, A. O. Hunt, 356.
Dental Degree in London University, 127.
Dental Faculties Association Criticised, L. Greenbaum, 143.
Dentinal Anesthesia, Thiesing Bonn, 43.
Dentifrices, Evil Effect, J. Turnbull, 122.
Empyemia of Maxillary Sinus, Geo. Zederbaum, 254.
Ethics, F. W. Harnden, 247.
Extension for Prevention, P. B. Noyes, 564.
Facial Neuralgia, Jas. E. Powers, 622.
Four Years vs. Three Years Course, F. L Platt, 135.

Helps in Practice Building, A. J. Flanagan, 115.
Hot Water as an Obtundent, S. H. Guilford, 87.
Identification of Iroquois Fire Victims, Geo. W. Cook, 141.
Inlay Matrix, C. C. Allen, 251.
In Sections or Otherwise, J. D. Patterson, 620.
International Dental Congress, Jas. Truman, 39.
Local Infection, R. J. Godlee, 74.
"Mary," R. E. Gilson, 504.
New Era in Dentistry, C. M. Wright, 625.
Nitrous Oxide Anesthesia, F. M. Richardson, 68.
Northwest vs. East, E. K. Wedelstadt and M. L. Rhein, 183.
Porcelain Crowns, F. E. Roach, 138.
Porcelain, What to do with it, W. T. Reeves, 60.
Pressure Anesthesia, R. B. Tuller, 186.
Prosthetic Porcelain, N. S. Jenkins, 512.
Pyorrhoea, H. C. Fitzharding, 518.
Replantation, Two Cases, J. H. Gibbs, 144.
Social Standing of Dentists, James Truman, 605.
Social Prejudice against Dentists, 515.
Swaged Shell Crown, W. A. Knowles, 82.
Toothsome Topics, R. B. Tuller, 33.
Useful Antiseptic, B. Bennette, 253.
We Made Better Dentists Then Than Now, D. R. Stubblefield, 20.
Why Porcelain Bridges Fail, Geo. W. Schwartz, 244.

### EDITORIALS.

Attendance at Dental Meetings, 144.
Called Meeting of National Faculties Association, 359.
Commercial Exhibitor as a Clinician, 149.
Consolidation of Dental Colleges, 196.
Daily Medical Journal, 148.
Dentistry as a Specialty of Medicine, 196.
Educational Situation, 520.
Exaggerated Statements, 44.
Examining Boards and Three-Year Course, 415.
Financing the International Congress, 416.
Four-Year Course, 358.
Fourth International Dental Congress, 476.
Honoring Old Graduates, 198.
International Dental Congress, 379, 418.
International Federation, 478.
Lectureship Vacant, 418.
Medical and Dental Training for Practice, 197.
Methods of Conducting Society Meetings, 577.
National Boards of Examiners, 477.
Pacific Coast Dental Congress, 627.
Pony for State Board Examinations, 257.
Porcelain as a Filling Material, 98.
Proceedings for Faculties Association for the Year 1904, 627.
Program of International Dental Congress, 408.
Social Standing of Dentists, 627.
Southern Branch of National Dental Association, 148.
Specialists, Is there a Call for Them? 313.
State Board Examination Questions, 257.
The College or a Higher Standard, 628.
The Three-Years Course, 415.
"Too Much Johnson," 313
William H. Whitslar Resigns Professorship, 360.

### ANNOUNCEMENTS.

American Dental Society of Europe, 99.
American Society of Orthodontists, 421.

Anderson, Indiana, Dental Society, 150.
Arkansas Board of Examiners, 581.
Army and Navy Dentists, 314.
California Board of Examiners, 99.
Canadian Dental Association, 317.
Central Michigan Dental Association, 316.
Central and Southwestern Michigan Dental Association, 151.
Chicago Dental Society, Officers, 259, 317.
Cleveland Dental Society, 45.
Committee on Organization of International Congress, 151.
James A. Dale, Editor, 581.
Dental Congress Banquet Announcement, 361.
Dental Journal Clearing House, 361.
Eastern Indiana Dental Society, 151, 317.
Excursion to St. Louis, 260.
Fraternal Dental Society, 45.
Federation Dentaire, 362.
Fourth International Congress, Local Committee Announcement, 261.
Gould's Medical Dictionaries, 260.
Harlan, A. W., Removal to New York, 361.
Hartford, Conn., Dental Society Officers, 580.
Harvard Alumni Association, 420.
History of S. S. White Dental Mfg. Co., 522.
Illinois Board of Examiners, 199, 204.
Indiana State Dental Society, 150, 261.
Institute of Dental Pedagogics, 630.
International Congress, Hotel List, 363.
International Dental Congress, 201, 318.
International Federation Program, 418.
Interstate Dental Fraternity, 361.
Journal of Oral Surgery and Medicine, 422.
Kentucky State Dental Association, 204.
Kentucky Board of Examiners, 580.
Michigan State Board of Examiners, 99, 151, 317.
Michigan State Dental Ass'n, 199, 258, 420.
Minnesota State Dental Association, 151.
Mississippi Valley Medical Association, 421.
Montana State Society Officers, 199.
National Association of Examiners, 100, 151.
National Association of Faculties, 258.
National Dental Association, 421, 479.
New Jersey State Dental Meeting, 99.
Northern Ohio Dental Society, 200, 259.
Ohio Board of Examiners, 259, 317, 480, 522.
Ohio State Dental Society, 45, 523, 581.
Patent Law Amendment, 360.
Pennsylvania State Board of Examiners, 260.
Physicians Visiting List, 269.
St. Louis Dental Society Officers, 99.
Taft, Dr. J., Memorial Resolutions, 45, 322, 370, 369.
Tennessee State Dental Association, 45.
The Dental Surgeon, 629.
Tri-State Committee of Organization, 420.
Wisconsin State Society Officers, 523.

### OBITUARY.

George H. Chance, 582.
J. Foster Flagg, 46.
Henry C. Howells, 422.
William D. Saunders, 152.
James R. Stuart, 152.
E. D. Swain, 320.
J. Taft, 205.
William W. Warren, 48.
T. B. Welch, 101.
Mrs. Sarah J. White, 526.
I. P. Wilson, 422.

### BIBLIOGRAPHY.

Bohm, Davidoff, Huber Histology, 523.
Burchards Pathology and Therapeutic, 579.
Donhan's Histology, 480.
Essig's Metallurgy, 480.
Irregularities of the Teeth, Talbott, 319.
Irregularities of the Teeth, Wallace, 366.
Organic and Physiological Chemistry, 100.
Warren's Pathology and Dental Medicine, 100.

### INDENTS.

Abortive Treatment of Boils, 423.
Action of Caries in Enamel, 565.
Adaptation of Partial Denture to Remaining Teeth, 6[illegible]4.
Adrenalin Dangers, 375.
Adrenalin Solution, 323,
Advanced Standing, 328.
After Pains from Extracting, 323.
Aid in Carrying Pumice, 50.
Alcohol in Porcelain Inlay Work, 264.
Aluminum Lining, 206, 215, 377.
Amalgam Formulae, 227.
Amalgam Inlay, 268.
Amalgam Mixing, 269.
Anesthesia, 529,
Anesthesia for Face Operations, 157.
An Interesting Item, 634.
Appendicitis from Diseased Teeth, 426, 584.
Applying Dam to Cervical Cavities, 155.
Areas of Immunity and Liability, 568.
Argurol, 633.
Artist and Artisan, 106.
Asymetry, 153.
Attaching Low Fusing to High Fusing Porcelain, 527.
A Veteran, 634.
Avoid Pain in Operating, 525.
Babbit Metal Dies, 376.
Bands in Regulating, 631.
Blessing, 506.
Boiling Point of Metals, 213.
Bridge Expedient, 210.
Bridging of Spaces in Soldering, 377, 530.
Broach in Tooth Affects Eyesight, 483.
Broken Facings Replaced, 372.
Building Dental Societies, 212.
Bur for Inlay Cavity Preparation, 373.
Camphenol-like Antiseptic, 249.
Camphor, 156.
Carbolic Acid Suicide, 486.
Case Hardening, 102.
Cavity Lines, 50.
Cheap Amalgam Fillings, 104.
Children Patients, 425.
Chiors Percha in Crowns, 482.
Clay Pipe Stems as Supports, 323.
Cleaning Impression Trays, 154.
Cleaning Teeth, 102, 589.
Cocainizing Teeth Through the Nose, 536.
Cocain and Morphine Abuse, 267.
Co-education in Dental Colleges, 428.
Coloring Cements to Match Teeth, 267.
"Combined Ray," 592.
Conduct of a Practice, 378.
Contouring Matrices, 50.
Corrosion of Silver Plates, 371.
Cotton Points, 50.
Counter Dies Made Quickly, 52.
Crowning a Dog's Tooth, 265.
Crowning Separated Root, 525.
Dangerous to be Safe, 583.
Death from Gas Administration, 590.
Defects in Educational System, 333.
Defects in our Professional Life, 325.
Delicacy of Manipulation, 583.
Dental Education Should be Begun Early, 334.
Dental Graduates, 264.
Dental Reciprocity, 161.
Dental Stage Properties, 208.
Dentition as an Indication of Age, 159.
Dentists in Germany, 323
Determining Factors in Extension, 564.
Diagnosing Devitalized Pulps, 631.
Diagnosing Pulp Sensitivity, 266.

Diagnosing for Plates, 532.
Doctors Called, 394.
Doctor and Society, 396.
Doctor as Diagnostician, 397.
Doctor and Self, 397.
Doctor and his Profession, 395.
Eau Dentifrice, 423.
Echafalta for Stomatitis, 211.
Editor and Doctor, 423.
Educational Demands of the Day, 327.
Empyema of Antrum, 427.
Endowed Colleges, 328.
English Dental Law, 160.
Erosion, 153.
Escharotic Tooth Medication, 53.
Eucaine, 54.
Evolution in Educational Standards, 327.
Exposure of Gold Bands, 206.
Expansion of Plaster, 632.
Extension Applied to Natural Defects, 567.
Filling Came Out, 539.
Filling the Matrix, 431.
Forma Percha, Bloir's, 264.
Formaldehyde in Milk, 210.
Four-Year Course a Necessity, 331.
Fusible Alloy, 207.
Gagging Overcome, 154.
Gauze Drains Contra-indicated, 482
Glossy Surface on Wax, 51.
Glycerite of Tannin for Sensitive Dentin, 372.
Guide for Fusing Porcelain, 485.
Gum Removal, 483.
Gum Retractor, 323.
Gutta Percha Plates, 215.
Harvard Dental School, 329.
Harvard's Situation, 329.
Harvard's Action Criticised, 330.
Heating Essential Oils in Teeth, 375.
Hickory Tooth, 526.
Hole in Gold Crown Repaired, 324.
Holocaine, 529.
Hydrogen Dioxide Criticised, 632.
Hydrogen Dioxide and Lime Water, 323.
Hydro-naphthol, 424.
Ideal in Dental Education, 333.
Immunity to Infection, 425.
Impression Taking, 266.
Impression Material, Prevent Adhesion, 483.
Improving the Dental Status, 206.
In Defense of a Tooth Saver, 107.
Influence of Practice on the Doctor, 398.
Injecting Pulps, 211.
Inlays without a Matrix, 156,
Instruct with Vigorous Sp.eech 527.
Investment Compound, 50.
Investment, 482.
Lactic Acid, 506.
Lead Washer for the Hypodermic Syringe, 631.
Light for Dark Days, 52.
Local Anesthetics, 633.
Low Fusing Enamels, 482.
Matrix Adaptation with Putty, 209.
Medical Instructors of Dental Students, 334.
Melanatic Cancer, 52.
Menthol Crystals, 633.
Method of Making a Gold Inlay, 63[illegible].
Migration of Metals, 371.
Modern Dental Outfit Dangerous, 484.
Moulding Softened, 264.
Mouth Alcohol Heater, 212.
Muscular Paralysis from Tooth Extraction, 268.
Nail Cleaning Liquid, 483.
Napkins, 505.
National Diploma, 583.
Natural Crown, 526.
Nature Teaches the Ideal Doctor, 401.
Neuralgia Remedies, 51.
Neuralgia Alcoholic Injection, 374.
Nenralgia and Anti-kamnia and Codeine, 270.
New Occlusal Lines for Artificial Teeth, 585.
Obtunding Dentine, 153, 265.
Odontolgia, 636.
Opening Pyrozone Tubes, 207.
Operative Field, 526.
Orthoform Cotton, 265.
Oxpara in Putrid Canals, 214.
Painful Dentition, 50.
Painless Pulp Removal, 371.
Palmer, Corydon, 534.

Pennsylvania Dental College Fire, 268.
Percentage Rule, 635.
Peroxide Dentifrice, 587.
Pink Rubber Fronts, 103.
Plaster Bowls, 153.
Plaster Impressions, 632.
Plastic Fillings, 155.
Platinum, 208.
Polishing Teeth, 533.
Polishing Vulcanite Plates, 264.
Poor Old England, 532.
Porcelain Restorations, 534.
Porcelain Teeth, Size of, 537.
Practice Building, 105.
Preliminary Training, 325.
Preparing an Impression Tray for Plaster, 637.
Pressure Anesthesia, 208, 376.
Preparing Roots for Logan Crowns, 376.
Preventing Drills Rusting, 337.
Prophylaxis, 531.
Pulp Removal, 53.
Pyorrhoea, 373, 482, 584.
Quick Pickle, 371.
Quick Separating contraindicated for Amalgam Filling, 484.
Radium, 158, 371.
Release Tight Forcep Joints, 374.
Removal of Crowns, 371.
Removal of Inlay Matrix, 373.
Roentgen Ray Dermatitis, 632.
Repairing a Crown, 375.
Repairing a Lower Plate, 154.
Report on Colleges, 575.
Restoring Tarnished Gold, 484.
Retaining Appliance, Simple, 636.
Retaining the Dam with Wire Ligatures, 157.
Return to Three-Years Course, 328
Root Canal Cleaning, 53.
Rubber Cement, 638.
Rust, Removal from Instruments, 372.
Salve for Cracked Hands, 267.
Saliva a Factor in Curing Oral Diseases, 485.
Sensitive Dentine, 51.
Septicemia from Alveolar Abscess, 103.
Setting Inlays, 525.
Shock, its Nature, 485.
Short Accounts, 50.
Shrinkage of Porcelain Overcome, 372.
Silence Aseptic, 154.
Skin Varnish, 483.
Sodium Dioxide as an Obtundent, 268.
Soft Water as a Cause of Caries, 207.
Soreness of Gum after Setting a Crown, 372.
Specialists, 326.
Sponge Gold, 324.
Split Roots, 528.
Sugar and Caries, 424, 430.
Sulphuric Acid and Ether for Canals, 530.
Sultan with Diamond Teeth, 633.
Supernumerary Teeth, 528.
Supporting a Split Root, 209.
Stovain as a Local Anesthetic, 588.
Swaging Caps to Roots, 153.
Systematic Treatment of Caries, 570.
Takes Money, 508
Taking the Bite, 587.
The Protective Role of Human Saliva, 637.
Tempering Instruments, 631.
Temporary Filling, 525.
Time Saved, 507.
Tooth Powders, 154, 374, 631, 635.
Tooth Resorption, 636.
Tooth Soap, 266.
To Renovate Hardened Mouldine, 632.
Training Children for Patients, 586.
Training for Dentistry, 326.
Tri-Chloracetic Acid, 324.
Use of Worn Mandrels, 324.
Using Tooth Powder, 637.
Value of Exercising the Teeth, 638.
Vacuum Chambers, 213.
Vaseline and the Second Impression, 528.
Veneering Vulcanite Plates, 269.
Vulcanite Shrinkage, 535.
Why the Rubber Dam, 269.
Wood Handled Instruments Undesirable, 265.

AUTOGRAPHIC.

Abell, B., 499, 502, 504.
Allen, C. C., 251.
Ambler, H. L., 306.

Atchison, B. B., 517.

Bachman, B., 372.

Badcock, J. H., 128.

Baldwin, A. E., 328.

Barber, W. A., 349.

Barnes, H., 391.

Beach, Dr., 266.

Bauman, J. B., 305, 352

Belcher, W. W., 212, 267, 378, 426.

Bell, B. F., 159.

Bennette, B., 253, 430.

Bennett, D., 586.

Bethel, L. P., 433.

Billmeyer, U. D., 53.

Bogue, B. A., 333.

Bonn, Thiesing, 43.

Bowling Tom, 638

Briggs, E. C., 329.

Broomel, Dr., 50.

Brophy, Truman W., 632.

Bumgardner, E., 193.

Burke, N. L., 155.

Byram, J. Q., 271, 306.

Callahan, J. R., 300, 389.

Champion, G. G., 133.

Cassidy, J. S., 403.

Chapple, Dr., 429.

Chase, R. L., 211.

Chittenden, C. C., 328.

Clemmer, J. W., 393.

Colyer, S., 424.

Colyer, F. J., 128.

Collins, F. C., 548, 554.

Combe, R., 426.

Conway, B. L., 206.

Cook, Geo. W., 141.

Crenshau, Wm., 526.

Crile, Geo. W., 157.

Cryderman, F. W., 113.

Cryer, M. H., 32,

Cudworth, W. H., 223.

Custer, L. E., 282, 311, 350.

Darby, E. T., 31, 590 .

Davis, C. L., 154.

Dawbarn, R. H. M., 425.

DeVries, Dr., 207.

Douglas, I., 502.

Eams, G. F., 325.

Eigel, G., 54.

Endleman, J., 375.

Essig, F. H., 467.

Farley, W., 107.

Fernandez, L. J., 373.

Field, F. H., 207.

Finlayson, T. W., 154.

Fitzhardings, H. C., 518.

Flanagan, A. J., 115.

French, E. C., 213.

Fowler, S. M., 468.

Fuller, E. S., 303.

Garhart, N. K., 217.

Gibb, J. H., 144.

Gilson, R. E., 504.

Godlee, R. J., 74.

Godon, Chas., 509.

Goslee, H. J., 285, 308.

Graber, R. L., 269.

Graham, D. M., 55.

Grant, J. S., 206.

Greenbaum, 143.

Griswold, E. R., 376.

Guilford, S. H., 30, 87.

Hadley, C. J., 51.

Hall, L. P., 470, 554, 640.

Hand, C. J., 553.

Harnden, F. W., 247, 372, 373.

Hartwig, 175.

Haskell, Dr., 376.

Hawley, C. B., 305.

Head, J., 528, 525.

Helmer, J. L., 264.

Hepburn, D., 131.

Hershey, G. S., 534.

Hetrick, Dr., 104.

Hewitt, A. C., 625.

Hildreth, C., 461.

Hines, P. F., 16, 462, 469.

Hodson, 50.

Hoff, N. S., 8, 242, 331, 461, 470, 503, 600.

Holden, R. S., 162.

Holloway, W., 210.

Honey, E. A., 601.

Howland, S. F., 153.

Howell, R. B., 338.

Hunt, A. O., 356.

Inglis, O. E., 264.

Jaboulay, Dr., 52.

Jackson, E. R., 335.

Jenkins, N. S., 512.

Jessel, A., 530.

Johnson, J. A., 211, 377.

Johnston, W. A., 54.

Jones, Dr., 390.

Joslin, F. W., 554.

Kay, W., 266.

Keenan, Wm., 588.

Kells, C. E., 209.

Kenney, J. B., 51.

Kettig, E. M., 533, 586.

Kirk, E. C., 27, 334, 379.
Kirk, H. M., 90.
Knowles, W. A., 82.
Lathrop, H. K., Jr., 461.
Lauderdale, C. F., 243.
Le Gro, B., 547.
Lee, B. H., 552.
Loeffler, E. T., 460, 490, 552.
Low, F W., 536.
La Selle, C. W., 215, 377.
Martin, O., 324, 371.
Martin, H. P., 109.
Marshall, J. S., 327.
Matheson, L., 130.
McCullough, P. B., 632.
McElhinney, M. G., 525.
McLean, W. T., 347, 351.
McNerthney, Dr., 428.
Miller, Dr., 637.
Mills, J., 53, 102, 153.
Mitchell, Wm., 375.
Montague, H., 133.
Moore, E. C., 502.
Morris, Dr., 103.
Moyer, S., 50, 482.
Munroe, Gr fton, 634.
Murphey, J. S., 584.
Noble, C. C., 487.
Noyes, F. B., 564.
Oakman, C. H., 163.
Ottolengui, R., 268.
Palmer, Corrydon, 534.
Palmer, L., 484.
Patterson, J. D., 183, 186, 620.
Peck, A. E., 594.
Platt, F. L., 135.
Price, W. A., 304.
Post, C. E., 208.
Powers, Jas. E., 622.
Prothers, J. H., 432.
Rafensperger, E. H., 528.
Raymond, H. C., 293, 304, 453.
Reclus, Prof. P., 589.
Reeves, W. T., 60, 222, 555.
Rhein, M. L., 52, 183, 217, 333, 526, 531, 533.
Ricard, D. S., 431.
Rich, A. C., 637.
Rich, John B., 634.
Richardson, 68.
Roach, F. E., 138, 217.
Robinson, W. J., 264, 270.
Rogers, C. E., 592.
Rohland, C. B., 525.
Rose, F., 160, 268.
Royce, E. A., 425.
Ruggles, S. D., 389.
Schamberg, M., 631, 636.
Scheurer, Dr., 324.
Schmitt, M. L., 584.
Schwartz, Geo. W., 244.
Sheehan, E., 50
Smale, M., 134.
Smith, H. T., 385, 392.
Smith, P. W., 265, 374.
Snyder, H. P., 469.
Soule, E. M., 268.
Sparks, R. E., 525.
Speer, G. B., 323.
Spence, S. J., 536, 587.
Spring, J. F., 461, 599.
Staples, Geo. S., 105.
Steel, J. F., 583.
Stephan, F. W., 153, 278, 531, 635, 637
Stephan, J. A., 351.
Stubblefield, D. R., 20.
Sweetnan, J. L., 502.
Talbot, W. O., 215.
Tawzer, Prof., 210.
Thompson, J. M., 482.
Thornton, A. W., 154.
Todd, W. H., 306, 390.
Thompkins, Dr., 373.
Thorpe, B. L., 634.
Tourtelatt, Dr., 374.
Truman, James, 28, 39, 330, 605.
Tuller, R. B., 186, 540.
Turnbull, J., 122.
Underwood, A., 134.
Vale, F. P., 485.
Waas, A. M., 153.
Wagner, J. F., 484.
Wallace, J. S., 366.
Waltz, A. J., 52.
Ward, M. L., 114, 243, 234, 232.
Watson, M. T., 468, 496, 503.
Watson, H. D., 223.
Wedelstadt, E. K., 183.
Whetslar, W. H., 504.
Wilson, Geo. H., 298, 392.
Windell, Dr., 636.
Worboys, C. H., 242, 552, 553, 595, 595
Wood, C. P., 461, 467, 470, 547.
Woods, J. A., 265.
Wright, C. M., 625.
Younger, Dr., 373.
Young, J. L., 471.
Zederbaum, Geo., 254, 541, 548.
Ziele, H., 95.

### ANNOUNCEMENTS.

The Cleveland Dental Society desires to extend its sincerest sympathy to the family of Professor Jonathan Taft. By his death we have lost a friend whose visits to our midst proved his greatness as an adviser because of his wisdom, sympathy, and undaunted courage to do right.

To the members of this society who were formerly his students the death of Professor Taft leaves a profound impression of his love and greatness. While now absent his life is before us an open book.

The society has therefore, resolved to send this statement of our love to Mrs. Taft and family and place these expressions upon our records.

W. H. Whitslar,
J. R. Owens,
Committee.

The officers elected at the last meeting of the Ohio State Dental Society are: For President, Dr. J. F. Stephan, of Cleveland; for Vice Presidents, Dr. W. T. McLean, of Cincinnati and Dr. H. L. Ambler, of Cleveland; for Secretary, Dr. C. D. Ruggles, of Portsmouth; for Treasurer, Dr. C. I. Keely, of Hamilton.

The Fraternal Dental Society of St. Louis, elected Dr. E. E. Hawstick, President; Dr. E. P. Dameron, Recording Secretary; Dr. J. A. Todd, Corresponding Secretary, and Dr. S. T. Bassett, Treasurer.

The 36th annual meeting of the Tennessee Dental Association was held at Lookout Inn, Chattanooga, Tenn., July 23d to 25th, 1903. Dr. W. H. Slater presided and the meeting was full of interest. There was a good attendance, many coming from other States. The attendance would have been larger but for the National meetings at Asheville near the same time. The president read a good paper on ethics, and there were ten other papers read before the meeting. Dr. A. A. McLanahan was superintendent of the clinics and they were well conducted. The porcelain inlay was much in evidence. Twelve new members were added, mostly young men. Dr. B. B. Bogle, of Nashville, was made President; Dr. J. T. Crews, of Jackson, Recording Secretary; Dr. W. K. Slater, of Knoxville, Corresponding Secretary; Dr. W. P. Sims, of Nashville, Treas.

The next meeting will be held at Jackson.

A. S. PAGE, Secretary.

